# Using computational modelling to reveal mechanisms of epigenetic Polycomb control

**DOI:** 10.1042/BST20190955

**Published:** 2021-02-22

**Authors:** Cecilia Lövkvist, Martin Howard

**Affiliations:** Computational and Systems Biology, John Innes Centre, Norwich Research Park NR4 7UH, U.K.

**Keywords:** computational models, epigenetics, mathematical modelling, polycomb repressive complex 2

## Abstract

The Polycomb system is essential for stable gene silencing in many organisms. This regulation is achieved in part through addition of the histone modifications H3K27me2/me3 by Polycomb Repressive Complex 2 (PRC2). These modifications are believed to be the causative epigenetic memory elements of PRC2-mediated silencing. As these marks are stored locally in the chromatin, PRC2-based memory is a *cis*-acting system. A key feature of stable epigenetic memory in cis is PRC2-mediated, self-reinforcing feedback from K27-methylated histones onto nearby histones in a read-write paradigm. However, it was not clear under what conditions such feedback can lead to stable memory, able, for example, to survive the perturbation of histone dilution at DNA replication. In this context, computational modelling has allowed a rigorous exploration of possible underlying memory mechanisms and has also greatly accelerated our understanding of switching between active and silenced states. Specifically, modelling has predicted that switching and memory at Polycomb loci is digital, with a locus being either active or inactive, rather than possessing intermediate, smoothly varying levels of activation. Here, we review recent advances in models of Polycomb control, focusing on models of epigenetic switching through nucleation and spreading of H3K27me2/me3. We also examine models that incorporate transcriptional feedback antagonism and those including bivalent chromatin states. With more quantitative experimental data on histone modification kinetics, as well as single-cell resolution data on transcription and protein levels for PRC2 targets, we anticipate an expanded need for modelling to help dissect increasingly interconnected and complex memory mechanisms.

## Introduction

Understanding the mechanistic basis of epigenetic memory is one of the most important questions in contemporary biology, with implications ranging from environmental response to cell differentiation. Epigenetic memory systems allow individual genes to remember a particular expression state that can then be maintained through subsequent DNA replication and cell division events, thereby permitting stable gene expression states vital for differential gene expression in different cell types.

One particularly important epigenetic system is the Polycomb system, capable of maintaining genes in a repressed state, mediated in part through Polycomb Repressive Complex 2 (PRC2). PRC2 catalyses the methylation of lysine 27 of histone H3 and constitutes a vital part of this *cis*-mediated epigenetic memory system [1]. PRC2 can bind to existing H3K27 methylation, become allosterically activated and then add more H3K27 methylation to nearby nucleosomes [2,3]. Such positive feedback is vital for memory maintenance to counter the inevitable dilution at DNA replication, where parental nucleosomes are diluted by (on average) a factor of two causing a consequent reduction in H3K27 methylation levels [4].

However, under exactly what circumstances such feedback can maintain persistent memory states was unclear. Such a lack of clarity is typical of many biological systems with multiple interacting components, often with positive and/or negative feedback, where the complexity of the system can rapidly outstrip our ability to intuit the underlying network behaviour. In such cases, mathematical modelling offers an attractive way forward, as it allows such complexity to be precisely encoded in a mathematical format. Analytical, or more commonly numerical approaches, can then be used to extract the behaviour of the system and make predictions. In the following sections, we will review the application of these techniques to epigenetic switching and memory, focusing specifically on the Polycomb system.

## Models for epigenetic memory with opposing histone modifications

**Figure 1.**
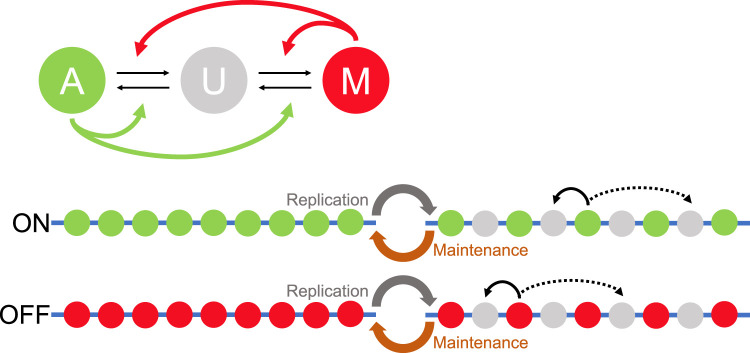
Memory model with opposing histone modifications. (Top) Schematic of the A–U–M model. A given histone has an activating (A) or a silencing mark (M) or is unmodified (U). The curved arrows represent self-reinforcing interactions where M histones promote transitions towards M (red arrows) and A histones promote transitions towards A (green arrows). (Bottom) The A–U–M model generates bistable states with histones predominantly either A (ON) or M (OFF). The states are maintained through replication despite the incorporation of new unmodified U histones at DNA replication. The self-reinforcing feedbacks can add marks to neighbouring histones (short-ranged, solid arrow) or, in addition, to distant histones (long-ranged, dashed arrow), thereby rebuilding the digital state that existed prior to replication.

An early model for *cis*-based epigenetic memory was developed in [5]. This model was originally introduced for the silenced mating-type locus of fission yeast, involving H3K9 methylation, before being adapted for use in the Polycomb system [6,7], see below. The model contains histones with an activating mark (A), a silencing mark (M) or which are unmarked (U) (Figure 1), and we therefore refer to it as the A–U–M model. To incorporate the known read-write feedbacks, a self-reinforcing mechanism was introduced, allowing an M-marked histone to add M marks to other U-marked histones. A related process was also incorporated allowing an M-marked histone to strip away A marks leaving a U histone behind (Figure 1). A symmetric set of assumptions was then introduced for reactions catalysed by A-marked histones (Figure 1), thereby forming a system of opposing, antagonistic histone modifications. Note that the model does not explicitly include the enzymes that perform the addition or removal processes, but rather assumes that each mark can indirectly catalyse these processes. Importantly, for appropriate parameter values, the model was able to maintain bistable states: an active state with mostly A-marked histones or a silenced state with mostly M-marked histones. In other words, a state initialised with mostly A's remains mostly A's, and similarly for a mostly M-state. Most importantly, these two states are stably maintained after DNA replication where the parental nucleosomes are randomly partitioned between the two daughter DNA strands and thereby diluted by roughly 50%. The strong positive feedbacks simply fill in the missing M's or A's after DNA replication to return the system to a digital, fully active or silenced configuration.

For bistability, the spatial range of the self-reinforcing feedbacks has an impact[5,8]. In the simplest long-range implementation, any given histone can interact with any other histone in the modelled region with equal probability. In this case, for appropriate parameter values, the model was bistable. On the other hand, for short-ranged interactions with a given histone only able to affect neighbouring histones, the states were found to be less bistable, due to the reduced overall cooperativity in the system. However, this lack of stability could be improved by allowing for more long-ranged interactions, as justified by the polymeric nature of the chromatin fibre and by the *in vivo* and *in vitro* behaviour of DNA contacts [9,10]. The number of possible marks (three for the A–U–M model) is also important for bistability [5]: the existence of the intermediate mark increases the scope for cooperativity and makes escape from a predominant M or A state difficult, increasing state lifetimes and thereby allowing for the existence of bistable states. Models with only two possible marks (say A and M) require extra and more restrictive assumptions to permit bistability. Analysis of this feature, as well as further explorations of generalised A–U–M type models can be found in [11,12]. Another model, appropriate for mouse embryonic stem cells, expanded the A mark into a full spectrum of H3K36me0/1/2/3 marks and M into H3K27me0/1/2/3 using differential equations [13].

Application of the A–U–M model to Polycomb dynamics has been within the context of cold-induced epigenetic silencing of the floral-repressor gene *FLOWERING LOCUS C* (*FLC*). Epigenetic memory is vital at *FLC* to ensure that flowering does not occur until after winter cold has passed [14]. Modelling proposed that silencing at *FLC* is digital, as appropriate for an A–U–M -like model, and therefore that an individual *FLC* locus does not change its expression state gradually: it is either ON or OFF and it is the proportion of OFF loci that increases with the duration of cold exposure [6,7,15]. In this way, at a whole plant level, expression of *FLC* falls according to the cold duration, while at each individual *FLC* locus, an all-or-nothing response is generated. Fluorescent imaging experiments in single cells in plant roots have successfully validated these ideas at *FLC* [15]. Furthermore, experiments with two-colour fluorescent labelling of the two different *FLC* gene copies [15,16] has provided strong evidence for independent memory states at each of the copies, thus favouring the *cis*-acting models put forward theoretically.

## Memory models with transcriptional antagonism

**Figure 2.**
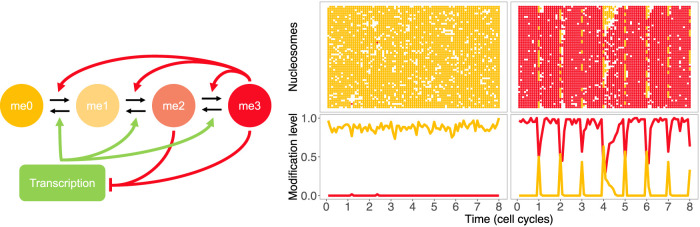
Memory model with transcriptional antagonism. (Left) Model with transcriptional antagonism to Polycomb silencing. H3K27me2/me3 feedback to generate more methylation (H3K27me2 feedback suppressed for clarity) and also silence expression. Transcription disrupts silencing through demethylation and nucleosome exchange. The overall module can also generate bistable gene expression states. (Middle, Right) Example model simulations showing H3K27 methylation dynamics over time. (Top) Each coloured marker shows the K27 state of an H3 histone in a system of 30 nucleosomes (60 histones) at a particular time. Red: me3; white: me1/2; orange: me0. The nucleosomes are initialised in me0 (middle) and me3 (right). (Bottom) Same simulations but showing overall H3K27me0 (orange) and H3K27me3 (red) levels. Sudden dips of H3K27me3 and rises of H3K27me0 caused by simulated DNA replication.

In the A–U–M model, the A-marks reflect a transcriptionally active state of the modelled gene, and it is these marks that antagonise the M-marks of the silenced state. However, another possibility is that it is the act of transcription itself that antagonises silencing, although A-marks could still modulate the transcriptional level and be added co-transcriptionally. Furthermore, at *FLC*, the most plausible A-mark, H3K36me3, was not found in a mirror image pattern to H3K27me3 (high H3K36me3/low H3K27me3 and vice-versa), as would be predicted by the most straightforward models [7]. Such a mirror pattern was instead only found in a small, so-called nucleation region downstream of the transcription start site, but not outside [17]. In a recent model, transcription was therefore explicitly included as the antagonist of silencing, with no active marks for simplicity, and including the full spectrum of H3K27 methylation marks (me0/1/2/3) (Figure 2), in the context of a mammalian Polycomb target [18]. In this model, the Polycomb read-write feedbacks are short-ranged, with bistability achieved in part by the effects of transcription occurring globally across the gene, and therefore being effectively long-ranged. The short-range PRC2 feedback also makes confining the silenced state much more straightforward: long-ranged PRC2 interactions can in principle lead to escape of silencing from specific domains by jumping over any potential insulator elements [18]. To feedback from the active state and antagonise silencing, transcription was assumed to promote demethylation and nucleosome exchange (as found, e.g. in [19]). The model was parameterised in part by data tracking histone modification levels as a function of time through the cell cycle [20], which led to the conclusion that H3K27 methylation dynamics were surprisingly slow, with H3K27me3 levels recovering throughout the cell cycle from dilution at the previous DNA replication event. This slow timescale might result from the need to buffer short-term fluctuations in transcription factor levels and thereby allow persistent silencing [18]. By including transcription in the model, it was also possible to explore the role of trans-factors and how the memory states are influenced by the externally driven gene-activation strength. The authors found that bistability could only be found within a range of gene-activation strengths [18]. The model is potentially therefore an important first step in marrying epigenetic memory dynamics with more conventional transcription factor control.

## Model generalisations for bivalency

Built into the fundamental structure of the A–U–M models is mutual exclusivity between the marks at each histone. Furthermore, dynamical antagonism between the marks creates bistable states with either M or A marks predominating at a given locus [5,11]. It is therefore in general difficult to generate configurations with substantial numbers of A and M marks at the same locus. However, experimentally, it has been observed that it is possible to generate such states, where it is believed that the opposing marks (e.g. H3K27me3 and H3K4me3) occupy different histone H3 tails on the same nucleosome or on adjacent nucleosomes, but rarely on the same histone [21–23]. It is clearly an important question to address whether generalised models can encompass such so-called bivalent states. To explore such bivalent chromatin, a model with a dramatically expanded number (144) of possible nucleosome modification states was employed [24]. The model included each possible nucleosome configuration and the read-write feedbacks (assumed to be long-ranged) observed in the literature. Despite a large number of possible states, the model was still found to be bistable with a preference for either silent or active marks, but where, for example, activating marks could still to some extent coexist with silencing marks on the same nucleosome [24], similar to the bivalency found experimentally.

## Models for epigenetic switching

So far, we have only considered models capable of maintaining epigenetic memory, assuming that the initial memory state has been correctly specified. However, it is also vital to understand how epigenetic memory states can be properly specified and switched. In this context, a recent extension of the A–U–M model considered Polycomb dynamics in the early Drosophila embryo [25]. The model included the dynamics of histone modifications at a Polycomb/Trithorax Response Element (PRE/TRE) and its coupling to a promoter. A PRE/TRE with silencing marks promoted the unbinding of a transcription factor from the promoter, while activating marks promoted binding. The state of the promoter can similarly feedback to the PRE/TRE, with a bound promoter enhancing stronger A-recruiting feedbacks and an unbound promoter enhancing stronger M-feedbacks. This model allows transcription factors during early development to switch and dictate the initial expression state which is then encoded in the epigenetic memory state of the PRE/TRE. The PRE/TRE can then enforce this specific expression state during subsequent development. In order for this process to function properly, the model predicted that the initial chromatin state at the PRE/TRE must be blank, so that it can be properly switched and specified by the promoter. This requirement could be ensured by the early very rapid cell cycles in Drosophila acting to regularly insert blank nucleosomes, and thus keep the PRE/TRE state naïve prior to promoter-determined specification [25].

Another important switching mechanism is the nucleation- and spreading-mediated silencing of heterochromatin in fission yeast [26]. To describe the dynamics of this switch, the A–U–M model was modified to include 3 short-range interactions and one long-range interaction (M promoting the transition from A to U). By allowing enhanced spontaneous additions of M in a nucleation region, the M marks accumulate there and are then spread out, thereby capturing the observed dynamics of nucleation and spreading. This modelling demonstrated a need for both long- and short-range interactions to recapitulate switching, spreading and maintenance of epigenetic states in fission yeast, with a need for short-range interactions to reproduce the sequential silencing of spatially distributed reporters [26]. Nucleation and spreading of H3K9 methylation in fission yeast was also modelled in [27], investigated using linear (short-range) and looping driven (long-range) interactions with a two-state model (U–M). The modelling again showed the need for long-range interactions to explain the experimental observations. An alternative hypothesis for spreading was explored in [28], where spreading (here of H3K27 or H3K79 methylation) from a nucleation region was modelled to occur through the movement of marked nucleosomes.

At *FLC*, Polycomb-mediated epigenetic switching also involves nucleation and spreading of H3K27me3. At *FLC*, the H3K27me3 nucleates in a region of ∼3 nucleosomes during cold treatment, the so-called nucleation region. During subsequent warm conditions, H3K27me3 spreads to cover the rest of the locus [29,30]. To model this within the A–U–M model, nucleation region histones were assumed to acquire a ‘competency' to nucleate with a probability that increases with time in the cold (implementing the effect of the upstream cold-induced protein VIN3 [31,32]). Competent states can then add M modifications more efficiently in the nucleation region, modifications which can subsequently flip the rest of the locus into a predominantly M state. The details of nucleation were further explored in [6], where it was concluded that the act of nucleation was likely to be an all-or-nothing digital event in order to be able to explain effective switching and silencing in fluctuating temperature environments. This prediction was subsequently confirmed experimentally through the identification of spreading mutants with normal nucleation but impeded spreading. In this case, silencing at a single-cell level, observed through fluorescent protein fusions, was still observed to be an all-or-nothing state [16]. Interestingly, however, memory in the spreading mutants was not stable and stochastic reactivation from silencing was observed at the single-cell level. This memory was nevertheless more persistent than would have been expected for histone marks in only a ∼3 nucleosome region. This finding has opened up the possibility that there may be additional memory elements of as yet unknown identity [16], a conclusion supported by analysis of mating-type genes in budding yeast, where epigenetic memory was surprisingly found to be independent of the number of nucleosomes involved [33].

## Higher-level state models

So far, all the models we have discussed have been mechanistic models at the level of individual histone marks. As we have seen, these models have successfully accounted for many of the switching and memory properties of epigenetic systems. However, another type of model which assumes the existence of certain memory states of entire loci, and then models switching between them, can also be informative. In these models, the dynamics of individual histone marks are not considered but only their collective overall state (e.g. silenced). Such a higher-level model was developed in the context of the mammalian immune gene *Bcl11b*, which is Polycomb-regulated and which controls T-cell fate commitment [34]. Using this system, it was tested experimentally using two-colour fluorescent labelling whether the two copies of the gene switch separately in cis, or together induced by a trans factor. In this case, the switch is to an active state, but to identify the type of switch, the authors modelled the two gene copies using a higher-level state model and compared with their experimental data. The scenario that could potentially explain the experimental observations was where a switch in trans and a switch in cis were both needed for gene activation [34]. Related higher-level models have also been developed for *FLC* in the context of temperature-dependent epigenetic state switching [35,36].

In [37], a synthetic biology approach was taken to manipulate epigenetic switching in a mammalian system. Various types of epigenetic memory modules were constructed experimentally, including Polycomb, which could be switched by applying an inducer, thereby recruiting appropriate epigenetic regulators to a target locus with a fluorescent reporter. To analyse these memory states, and to help study the switching timescales of various types of epigenetic memory, a higher-level state-switching model was introduced. The model included stochastic switching between three states: active, reversibly silent and irreversibly silent. By comparing the model to the controlled switching in the experiments, kinetics of the transitions between the three states could be extracted. The authors also observed that long-lasting silencing (and reactivation) in the Polycomb system occurs through stochastic all-or-none events, similar to observations at *FLC* and *Bcl11b*.

## Modelling within the 3d chromatin environment

Up to this point, none of the histone-level models we have discussed specifically includes details of the local 3d configuration of the chromatin. Instead, effective histone interaction rules were employed, for example allowing all histones to interact with all others within a specified region or following a power law decaying interaction strength with distance [5]. Other models have sought to examine how much can be explained purely by local interactions between nearest neighbour histones, finding that robust bistability is still possible and therefore that longer-ranged interactions are not a prerequisite for stable memory states to exist [18]. However, it is clearly important to imbed these models in a more realistic chromatin environment, fully incorporating the 3d nature of the DNA polymer fibre. Discussing these issues comprehensively is, however, beyond the scope of this review, and we therefore refer the reader to other recent reviews and primary literature [38–41].

## Perspectives

- Epigenetic interaction networks are sufficiently complex that intuition is insufficient to dissect their behaviour. Computational modelling can rigorously assess these behaviours and predict new features.
- Current models focus on mutual antagonism between silenced and activated states with self-reinforcing feedbacks, generating bistable gene expression states. Switching can often be achieved by controlled nucleation of silencing in designated nucleation regions, with nucleation also being an all-or-nothing event.
- With increasing data on single-cell dynamics of Polycomb targets, there will be a continued need to refine and supersede existing models to help provide deeper mechanistic understanding. New concepts such as phase separation may need to be incorporated into the modelling, resulting, for example, from the potential intrinsic phase separating properties of chromatin [42].

## Abbreviations

FLC: FLOWERING LOCUS C
PRC2: Polycomb Repressive Complex 2
PRE/TRE: Polycomb/Trithorax Response Element
